# Investigation and Feasibility of Combined 3D Printed Thermoplastic Filament and Polymeric Foam to Simulate the Cortiocancellous Interface of Human Vertebrae

**DOI:** 10.1038/s41598-020-59993-2

**Published:** 2020-02-19

**Authors:** William Clifton, Mark Pichelmann, Alexander Vlasak, Aaron Damon, Karim ReFaey, Eric Nottmeier

**Affiliations:** 1 Department of Neurological Surgery, Mayo Clinic Florida, Jacksonville, FL USA; 2 Department of Education, Mayo Clinic Florida, Jacksonville, USA; 3 Department of Neurosurgery, Mayo Clinic Health Systems, Eau Claire, WI USA

**Keywords:** Physiology, Anatomy, Medical research

## Abstract

Disorders of the spine are among the most common indications for neurosurgical and orthopedic surgical interventions. Spinal fixation in the form of pedicle screw placement is a common form of instrumentation method in the lower cervical, thoracic, and lumbar spine. A vital principle to understand for the safe and accurate placement of pedicle screws is the palpable difference between the cortical and cancellous bone, both of which have different material properties and compositions. Probing and palpation of the hard cortical bone, also known as the “ventral lamina”, covering the neural elements of the spinal canal during screw placement provides manual feedback to the surgeon, indicating an impending breach if continued directional force is applied. Generally, this practice is learned at the expense of patients in live operating room scenarios. Currently, there is a paucity of human vertebra simulation designs that have been validated based on the *in vivo* ultrastructure and physical properties of human cortical and cancellous bone. In this study, we examined the feasibility of combining three-dimensionally printed thermoplastic polymers with polymeric foam to replicate both the vertebral corticocancellous interface and surface anatomy for procedural education.

## Introduction

Back pain from spinal disorders is one of the most common diagnoses in medicine^1–3^. The number of spinal surgeries has significantly increased over the last decade, and spinal fixation in the form of pedicle screw placement is a common procedure for lower cervical, thoracic, and lumbar instrumentation^4^. Mastering pedicle screw insertion techniques is a vital component of both neurosurgical and orthopedic training programs^5,6^. Safe pedicle screw placement revolves around a comprehensive knowledge of pedicle anatomy in relation to the surrounding neurovascular structures^7^. Case volume and quality among training programs are highly variable both in the United States and the world, which can significantly affect exposure and competency regarding these techniques^8–10^. Two common adjuncts to surgical educational curricula include cadaveric models and simulation^11–20^. The use of cadaveric tissue is fraught with variability in specimen quality, accessibility, and cost^21–23^. In addition, many institutions are not able to facilitate human tissue specimens due to complex housing and personnel requirements for human tissue storage. In order to mitigate these limitations, simulation has become a popular method of alternate surgical education. Three-dimensional (3D) printing has been utilized for accurate replication of spinal anatomical features^24–30^. Multiple material printing, polyurethane injection molds, and virtual reality programs have also been investigated for replication of the corticocancellous interface for pedicle screw insertion training^16,31^. Other studies have investigated varying thermoplastic filament infill percentages to provide a palpable difference during simulated cancellous access^32^. Although biomechanical investigations have been explored on these single material models, replicating the granular details of cortical and cancellous material properties and composition has had limited investigation. There is significant variability in the histologic microstructure of cortical and cancellous bone, especially in the porosity index, which is considerably higher in cancellous bone^33–38^. Polyurethane foams have been investigated with regards to biomechanical properties analogous to human vertebrae, however this has mainly been performed with a single foam material and with injection molding processes that have considerably less ability to replicate patient and disease specific anatomical features of vertebral elements than 3D printing^31,39^. To our knowledge, there has not been an investigation of the feasibility of combining multiple polymeric materials with 3D printing techniques to replicate the ultrastructure of vertebral bone. Our hypothesis for this study was that combining 3D printed thermoplastic vertebral model shells and polymeric foam would be a feasible methodology for simulating corticocancellous bone. The compatibility of these unique models with standard spinal surgical instruments and instrumentation methods were also investigated, along with the cost of model production.

## Methods

### Materials

In this feasibility experiment, we utilized 3D printed additive manufacturing techniques to produce the vertebral models. An Ultimaker S5 Dual Extrusion 3D printer (Ultimaker; Utrecht, Netherlands) was used to produce all 3D printed materials. This is a desktop Fused Deposition Modeling (FDM) 3D printer with total dimensions of 495 × 457 × 520 mm that is commercially available for ~$4999.99. Specifications include a dual printer head for a two-nozzle system which allows simultaneous multiple material printing, a XYZ build volume of 330 × 240 × 300 mm, XYZ resolution of 6.9 × 6.9 × 6.9μm, and maximum build speed of 24 mm^3^/sec. The two thermoplastic filaments investigated for feasible compatibility with the polymeric foam were acrylonitrile butadiene styrene (ABS) and polylactic acid (PLA). These were chosen due to their comparable material properties with human cortical bone as previously investigated by Bohl, *et al*. and Hao, *et al*.^28,29,40^. The 2.85 mm diameter ABS filament had a melting temperature range of 225–245 °Centigrade (C), tensile modulus of 2,030 MPa, 34% elongation at break, Shore D hardness of 76, melt mass-flow rate (MFR) of 41 g/10 min at 260 °C, density of 1.04 g/cm^3^. The 2.85 mm PLA filament had a density of 1.25 g/cm^3^, melting temperature of 45–160 °C, tensile modulus of 2,346.5 MPa, 5.2% elongation at break, Shore D hardness of 83, MFR of 6.09 g/10 min at 210 °C.

### Polymeric foam production and thermal investigation

Polymeric foam was used to investigate the compatibility with thermoplastic polymers to represent the corticocancellous interface. Polymeric foam has been shown to be a useful method of recreating trabecular bone due to its porosity and density properties which can be manipulated based upon polymerization environment^41–43^. The foam components were acquired in a two-part mixture (Parts A & B) directly from the manufacturer (Smooth-On; East Texas, PA). Part A consists of a proprietary mixture of 4,4′ methylene bis (phenylisocyanate), benzene, 1,1′-methylenebis[4-isocyanato-], and methylenediphenyl disocyanate. Part B contains a proprietary aqueous surfactant mixture that catalyzes polymerization of Part A to polyisocyanate with chemical foaming when mixed in a 1:1 ratio and stirred for 30 seconds. The foam becomes porous due to chemical blowing from carbon dioxide byproducts that create microscopic and macroscopic cavities within the polymeric product. The mixed components have a pot life of 90 seconds before curing begins, with full cure time ~120 minutes. Expansile volume is 400%, with a density of 0.25 g/cm^3^ after full cure, which is similar to human cancellous bone^44^.

Combining Part A and Part B produces an exothermic polymerization reaction^45^. The exothermic nature of the reaction limits the ability combine the foam with thermoplastic polymers if the reaction temperature reaches the chosen thermoplastic’s melting point, which would distort the architecture of the 3D printed models. Investigation of the standalone baseline and maximum temperature range during polymerization was performed using a digital thermometer (Taylor LED Stem Thermometer, Taylor Precision Products; Oak Brook, IL) with a temperature range of −40 to 232 °C. Twenty (20) ml of Part A and 20 ml of Part B was placed into a 50 ml plastic container and mixed vigorously for 30 seconds as per the manufacturer. Initial temperature readings (T_0_) were recorded, and temperature documentation was recorded at 2-minute intervals until maximum temperature (T_max_) was reached. T_max_ was defined as the maximum temperature recorded in the polymerized foam before a decrease in temperature was identified.

### Vertebral model production

With institutional IRB approval, a CT (computed tomography) scan of an adult patient with 1-millimeter (mm) slice thickness was acquired from an anonymized, encrypted institutional database which does not record identifiable patient information. Patient anonymized DICOM (Digital Information and Communications in Medicine) data is deposited into the database after signed consent, and unable to be linked to identifiable information. The completely anonymized DICOM files were downloaded onto an encrypted hard drive and reviewed for inclusion criteria. CT reviewing was performed by three individuals (WC, AD, KF) on 3D Slicer (Slicer, v. 4.10.2, 2018). Criteria for inclusion of DICOM data were: inclusion of a complete vertebral column in the study, no surgical spinal instrumentation present on the CT, and no traumatic or iatrogenic deformity of the native anatomy (including previous laminectomy or disruption of posterior elements). Slice-based thresholding was then applied to the CT DICOM files with a range of 193–3000 Hounsfield Units. This particular range established the boundaries of the desired vertebrae in this particular DICOM data set. The vertebral interfaces were manually segmented in each individual CT slice in order to establish maximum accuracy of the vertebral bony associations. The thresholding and segmentation processes were performed by two individuals (WC and AD) with equally divided data sets, and quality and inclusion of thresholded anatomical structures was inspected on each slice by the opposite individual. Both individuals have extensive experience in thresholding and segmentation of anatomical structures using 3D Slicer, and independent assessment of data sets was performed to minimize observer bias. This workflow in combination with 1 mm DICOM CT slice thickness has been shown to be a highly accurate means of recreating spinal anatomic features with 3D printing^46^. The finalized selections were inspected in a three-dimensional projection within 3D Slicer, rendered to STL (Standard Tessellation Language) format, and then edited for manifold assurance using Meshmixer (Autodesk, 2017). Cura (v.4.0, Ultimaker, Netherlands) software was used for slicing and production of the models. The STL files are loaded onto the virtual software platform in Cura and spatially arranged for maximum printer efficiency. A 1 mm outer shell with 0% infill is used to produce hollow models that can be filled with the polymeric foam. We chose a 1 mm shell in order to replicate the average thickness range of cortical bone in the human vertebrae^44^. ABS filament at a diameter of 2.85 ± 0.05 mm is printed through a 0.4 mm nozzle at a bed temperature of 80 °C, nozzle temperature 250 °C, and nozzle extrusion speed of 70 mm/sec. PLA filament at a diameter of 2.85 ± 0.05 mm is printed through a 0.4 mm nozzle at a bed temperature of 80 °C, nozzle temperature 200 °C, and nozzle extrusion speed of 70 mm/sec. ABS and PLA cost expenditure is approximately $0.02 per gram or $0.46 per meter.

### Combining 3D printed models with polymeric foam

The hollow 3D printed vertebral models are secured and a 5 mm × 5 mm opening is drilled in the anterior portion of the vertebral body. The total volume of the desired vertebral model(s) was calculated using a volume rendering and analysis module within Meshmixer. The total volume is rounded to the nearest cm^3^, and divided by 4 in order to determine the amount of initial liquid foam mixture to be inserted into each model for complete filling of simulated cancellous bone without excessive foam spillage and distortion of the printed model external features. Part A and Part B of the foam reactants are mixed in a 1:1 ratio with a total volume equal to ¼ of the total volume of the 3D printed models in order to account for the 400% increase in volume after complete curing, where:

$${\rm{Part}}\,{\rm{A}}\,({\rm{ml}})+{\rm{Part}}\,{\rm{B}}({\rm{ml}})=({\rm{Total}}\,{\rm{Calculated}}\,{\rm{Volume}}\,{\rm{of}}\,3{\rm{D}}\,{\rm{Printed}}\,{\rm{Model}})/4$$

The calculated volume is injected into the vertebral model through the drilled hole with a standard syringe, and allowed to set for the complete cure time of 2 hours before use. Ambient conditions are in the range of 20–23 °C during curing with 40–60% humidity, inside a facility with air exchange protocol of 23 times per hour in order to limit inhalation of gaseous byproducts^47^. After the allotted cure time, the models are inspected for any deformities or anatomical distortions from the exothermic polymerization of the polyisocyanate foam. Excess foam is trimmed with standard diagonal cutting pliers.

## Results

**Figure 1:**
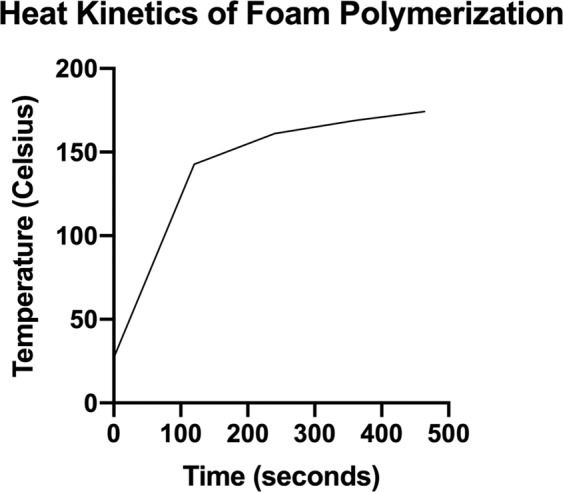
Heat kinetics of polyisocyanate foam polymerization. T_max_ = 174.2 °C at 464 seconds cure time.

We began by selecting a two-part porous polyisocyanate foam which has a complete cure density value range of 0.25 g/cm^3^, which falls within the density range of human cancellous bone in radiographic and cadaveric studies^48^. This foam creates a strong exothermic reaction during polymerization, thus limiting the compatibility and number of feasible thermoplastic material combinations^45^. In order to investigate the exothermic properties of the selected foam during the polymerization process, 20 ml of Part A and 20 ml of Part B were mixed together in a 50 ml open container with digital recording of temperature changes. The maximum temperature recorded was 174.2 °C during this initial test. The results are demonstrated in Fig. 1.

We selected two inexpensive and commonly used thermoplastic polymer 3D print filaments to simulate the cortical vertebral bone “shell”: polylactic acid (PLA) and acrylonitrile butadiene styrene (ABS), which have densities of 1.25 g/cm^3^ and 1.04 g/cm^3^, respectively. Their Shore D hardness values are 76 and 83, respectively. These filaments were chosen for their hardness and density values which fall in the range of previously investigated radiographic and cadaveric human cortical bone measurements^34,49,50^.

**Figure 2:**
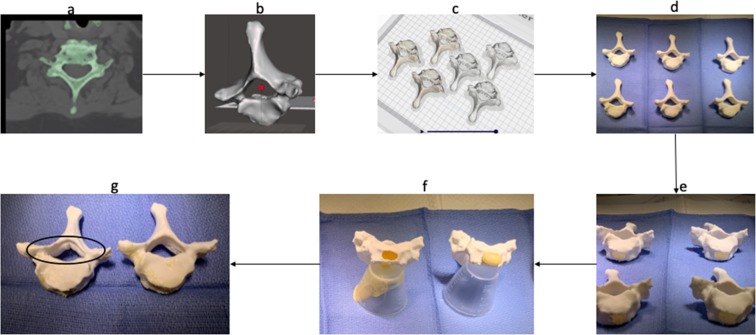
Workflow of DICOM (**a**) to STL (**b**,**c**) to ABS/PLA vertebral model production (**d**,**e**) and feasibility of combination with polymeric foam (**f**). The PLA model showed anatomic integrity failure (black circle) after foam injection due to the exothermic polymerization reaction (**g**), whereas the ABS model did not change in external shape.

The compatibility of the polymeric foam within 3D printed vertebral models was tested. In order to perform this, anonymized DICOM (Digital Imaging and Communications in Medicine) files were acquired through encrypted institutional software. A C7 vertebral STL file was created for initial polymer compatibility testing. This particular vertebra was chosen as an initial test model due to its complex anatomical features and small pedicle dimensions relative to other human vertebrae, which would require complete retention of external anatomic fidelity after foam insertion in order to use successfully as an educational tool. Six identical C7 vertebral STL files were uploaded into the slicing software and printed successfully on a dual extrusion (multi-material) desktop FDM (Fused Deposition Modeling) printer (see Fig. 2).

Each individual STL file volume was calculated using volumetric analysis in Meshmixer software 18.65 cm^3^. Two 0.4 mm diameter nozzles were used for PLA and ABS filament extrusion simultaneously to produce three PLA and three ABS C7 vertebral printed models for the feasibility study. The 3 PLA models were printed at a nozzle temperature of 200 °C, bed temperature 80 °C, and nozzle speed of 70 mm/sec. The three ABS models were printed at a nozzle temperature of 250 °C, bed temperature 80 °C, and a nozzle speed of 70 mm/sec. Total print time for production of models was 18 hours and 4 min. Total PLA material consumption was 4.59 meters (36 grams), and total ABS material consumption was 4.06 meters (28 grams).

The C7 vertebral models were divided into two groups: an ABS group and PLA group according to the material properties. A 5 mm × 5 mm hole was drilled in the anterior portion of the vertebral body to gain access to the inner portion for filling with the foam liquid mixture. A total of 5 ml of combined Part A and Part B were injected into each individual PLA and ABS C7 models according to volumetric calculations to fully accommodate the 400% increase in volume at full cure. The models were allowed to fully cure for 120 minutes per manufacturer specifications, and each model was inspected for any anatomic deformity that had occurred during the foam curing process. The three ABS models demonstrated no deformity after full cure time. The three PLA models demonstrated significant anatomic deformation which compromised model anatomic integrity (see Supplementary Material Video 1). This observed result was consistent with our pre-combination thermal recordings of exothermic foam polymerization, which greatly exceeded the melting temperature range of PLA.

**Figure 3:**
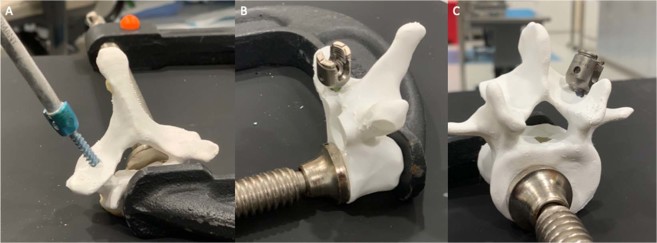
Instrumentation and validation of combined material (**A**) C7 vertebral models with 4.0 × 26 mm screws, (**B**) T6 vertebral models with 6.0 × 45 mm screws, and (**C**) L5 vertebral models with 8.0 × 50 mm screws.

In order to assess compatibility with standard spine surgical equipment and instrumentation and validate the combined polymer models for simulation of different vertebral levels, sixty ABS vertebral models of representative cervical, thoracic, and lumbar levels (20 C7, 20 T6, and 20 L5) were produced using the same software and additive manufacturing process. These vertebral models were chosen due to their representative structural architecture of pedicle and posterior element anatomy for the cervical, thoracic, and lumbar spine. The printed hollow models were filled with polyisocyanate liquid foam according to volumetric proportions as previously described and allowed to fully cure. A board-eligible neurosurgical spine fellow (WC) with over 1000-case experience in spinal surgery and instrumentation performed pedicle screw insertion of each vertebral model using standard freehand technique (see Supplementary Material Video 2). A total of 120 pedicle screws were placed in the ABS/polymeric foam models. The C7 models were instrumented with 4.0 × 26 mm screws, T6 models with 6.0 × 45 mm screws, and L5 models with 8.0 × 50 mm screws (see Fig. 3).

Models were assessed for integrity after pedicle probing, tapping, and screw placement. Model failure was defined as breaking, splitting, or cracking of the model during standard instrumentation. The results are listed in Table 1.

**Table 1:** Vertebral model integrity rate after instrumentation.

Vertebral Model	Pedicle Screw Size	Number of Pedicle Screws Inserted	Model Integrity Rate
C7 (n = 20)	4.0 × 26 mm	40	100%
T6 (n = 20)	6.0 × 45 mm	40	100%
L5 (n = 20)	8.0 × 50 mm	40	93%

There were no model failures during pedicle probing or tapping. There were 3 pedicle breakages during instrumentation of L5 vertebral models due to technique error (screws placed laterally in each of the three instances), otherwise there were no model failures during pedicle screw placement. The cost of each combined material vertebral model was calculated by adding the cost of ABS material use (~$0.02/g) to the cost of liquid polymeric foam (~$0.03/ml) The cost of each model production is listed in Table 2. Model costs were inexpensive. The L5 vertebral model cost was twice that of C7 and T6 due to the larger vertebral size and increased volume of liquid foam required, but still remained much less than $1 in total production cost.

**Table 2:** Cost, material usage, and production time of individual combined-material vertebral models.

Vertebral Model	ABS Material Use (g)	Print Time per Model (hr:min)	Liquid Foam Use (ml)	Estimated Cost per Combined Material Model ($)
C7	7 g	2:46	5 ml	$0.29
T6	7 g	2:45	5 ml	$0.29
L5	15 g	4:12	10 ml	$0.60

## Discussion

The results of this study indicate that the heat generated from the chemical polymerization of polyisocyanate foam exceeds the melting range of PLA, thus limiting compatibility for material combination for accurate anatomical model of external vertebral features. ABS has a much higher melting point range than PLA, but also falls within the density range of human cortical bone^34^. ABS has also demonstrated comparable haptic feedback to human cortical bone during drilling exercises and objective measurements^40^. ABS is a viable choice for FDM filament use in creating 3D printed vertebral shells for combination with polymeric foam to replicate the corticocancellous interface. This is applicable for cervical, thoracic, and lumbar 3D printed models according to our validation through instrumentation and screw placement in representative anatomical prototypes. Polymeric foam has been previously investigated to simulate cancellous bone for radiographic and surgical education purposes^51,52^. Polyurethane, porous bone cement, and low-density polyethylene have also been used to replicate the mechanical and material properties of trabecular bone^53^. The cortico-cancellous interface has also been simulated using polymeric foam combined with carbon fiber reinforced epoxy to provide a two-material model for surgical and biomechanical demonstration, and have been shown to adequately replicate the mechanical properties of human bone^54^. Despite the mechanical fidelity of these models, the accuracy of using injection molding processes alone for replication of the fine details of surface anatomy for the human spine has been shown to be inferior to rapid prototyping techniques^24^. By combining the accuracy of 3D printing with the mechanical and material properties of thermoplastic filament and polymeric foam, the advantages of both materials can be used to create an innovative simulation for spinal instrumentation.

**Figure 4:**
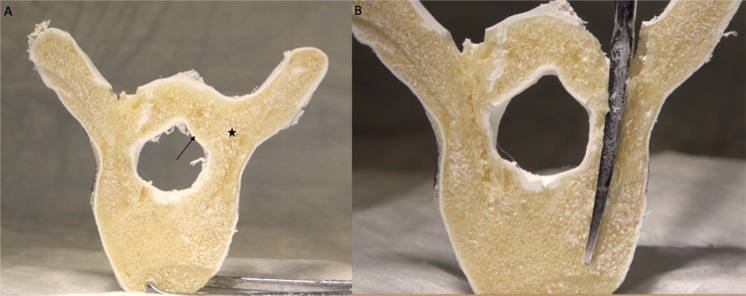
Cross-section through a combined 3D printed ABS/injected polyisocyanate foam vertebral model. (**A**) The foam generates a lower density, porous infill (star) compared with the thermoplastic “cortical” bone (arrow). (**B**) A curved pedicle probe can be inserted into the porous foam matrix in the same manner as *in vivo* for creation of a safe pedicle trajectory into the vertebral body.

**Figure 5:**
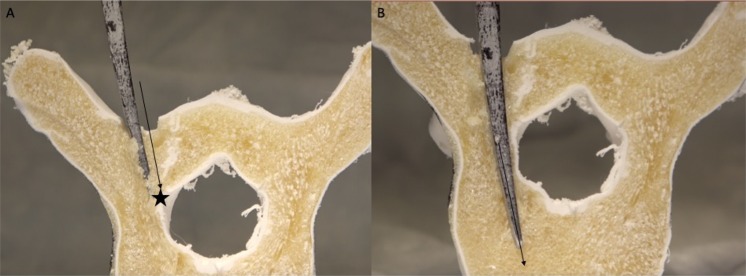
Demonstration of learning the ventral lamina concept. (**A**) The higher density ABS 3D printed thermoplastic outer boundary of the spinal canal (star) can be palpated during pedicle probing, simulating cortical bone. (**B**) The angle of pedicle probe insertion can then be adjusted (black arrow) to fall within the less dense simulated cancellous bone in order to avoid a breach into the spinal canal. This biomimetic model provides a reproducible and potent learning tool for spine surgical trainees to understand haptic principles of vital spine surgical techniques.

The ability to easily and cost-effectively combine multiple polymeric materials with extrinsic and intrinsic properties analogous to representative anatomical structures has important implications for orthopedic and neurosurgical training, biomechanical investigations, and instrumentation assessment. Desktop FDM 3D printing is cost-effective, easy to implement in an education program, and has been shown to provide highly accurate (micron level) detail of external anatomical features^55^. However, the singular use of this technology to recreate the histological material properties of human vertebrae falls short in limited material selection as well as feasibility for multiple material combinations^28^. This printing method has been explored in previous investigations, with some promise of biomechanical comparability to human vertebrae^24,28,56^. However, the porosity of the matrixed infill and the physical material specifications of simulated cancellous bone have not currently been able to be accurately replicated with thermoplastic 3D printing alone. Polyurethane foams have been shown to accurately replicate cancellous bone both in material properties and in porous structural composition^57^. This experiment showed that by combining ABS 3D printed thermoplastic filament with porous polymeric foam, an anatomically and structurally accurate vertebral model for demonstration and practice of spinal instrumentation skills can be constructed (see Figs. 4 and 5).

During pedicle probing, the trabecular and cortical bone interface is accessed for demonstration of the cancellous channel in which the posterior spinal elements connect to the vertebral body. An important anatomical concept to understand for safe screw placement, first described by Lehman, *et al*. is the “ventral lamina”, or the cortical bone covering the spinal canal and contiguous with the medial pedicle wall^58^. The ventral lamina is composed of dense cortical bone and can be felt during pedicle probing and screw insertion in order to gauge the limits of the pedicle boundaries. Recognition of this landmark is vital for safe placement of pedicle screws, as identification of the dense cortical bone during pedicle probing gives palpable feedback to the operator, in which further insertion of the instrument may result in pedicle violation. The haptic feedback of ventral lamina palpation is classically learned through experience in residency or fellowship training programs in operating scenarios on live patients^16,59,60^.

The costs associated with graduate surgical education has continued to rise over the last decade^61^. Dedicated curricular adjuncts to live operative exposure have been implemented, but are significantly limited by high cost and facility regulations^18–20^. Orthopedic surgical techniques, with spine surgery in particular, require significant hands-on learning time in order to gain a three-dimensional understanding of operative anatomy and manual feedback during various surgical scenarios. We have shared our detailed methods of production for these multi-polymer models in order to provide institutions with a stepwise means of creating multi-polymer anatomical models for education of spinal surgical techniques. It is likely that this method of polymer combination may also be applied for simulation of other orthopedic procedures and anatomical structures (long bones, digits, etc.) that require demonstration of the corticocancellous interface for fidelity^62^.

## Conclusions

This translational study demonstrated that combining 3D printed ABS vertebral models with porous polyisocyanate foam is a feasible, cost effective, and valid method of simulating the corticocancellous interface of human vertebral bone for surgical education of spinal instrumentation methods. To our knowledge, this is the first study to investigate thermoplastic polymer combination for spinal surgical simulation of the ventral lamina and corticocancellous bone. The application of material properties with accurate representation of vital anatomic structures can be used to create powerful and cost-effective educational tools for surgical training, while simultaneously maximizing patient safety.

### Ethics statement

The present study was carried out in accordance with the relevant guidelines and regulations/ethical principles of the Declaration of Helsinki.

## Supplementary information

Supplementary Video 1

Supplementary Video 2
